# Online resources as a source of information for exercise and physical activity in solid organ transplant recipients

**DOI:** 10.3389/fspor.2024.1353663

**Published:** 2024-04-30

**Authors:** Tania Da Silva, Rozhan Momen, Noor Al Kaabi, Muhib Masrur, Sandra Holdsworth, Karina Prevost, Sherrie Logan, Daniel Santa Mina, Istvan Mucsi, Mamatha Bhat, Ana Carolina Alba, W. Darlene Reid, Manoela Ferreira, Sunita Mathur, Tania Janaudis-Ferreira, Lisa Wickerson, Dmitry Rozenberg

**Affiliations:** ^1^ Temerty Faculty of Medicine, University of Toronto, Toronto, ON, Canada; ^2^ Toronto General Hospital Research Institute, University Health Network, Toronto, ON, Canada; ^3^ Faculty of Kinesiology and Physical Education, University of Toronto, Toronto, ON, Canada; ^4^ Institute of Medical Science, University of Toronto, Toronto, ON, Canada; ^5^ Canadian Donation and Transplantation Research Program, Edmonton, AB, Canada; ^6^ Ajmera Transplant Program, University Health Network, Toronto, ON, Canada; ^7^ Peter Munk Cardiac Centre, Ted Rogers Centre for Heart Research, Toronto, ON, Canada; ^8^ Department of Physical Therapy, Temerty Faculty of Medicine, University of Toronto, Toronto, ON, Canada; ^9^ School of Physical and Occupational Therapy, McGill University, Montreal, QC, Canada; ^10^ Respiratory Epidemiology and Clinical Research Unit, Centre for Outcomes Research and Evaluation, Research Institute of the McGill University Health Centre, Montreal, QC, Canada; ^11^ Division of Respirology, Temerty Faculty of Medicine, University Health Network, University of Toronto, Toronto, ON, Canada

**Keywords:** exercise, physical activity, solid organ transplantation, internet resources, health education

## Abstract

**Introduction:**

Exercise training post-transplant has been shown to improve physical function and quality of life in solid organ transplant (SOT) recipients. Online resources in the form of websites and videos are commonly used to provide education and instruction on exercise and physical activity in SOT; however, the content and quality of these online resources has not been evaluated.

**Methods:**

The first 200 websites and videos identified on Google and YouTube using the English search term “exercise and physical activity in solid organ transplantation” were analyzed. Website and video content was evaluated based on 25 key components of exercise and physical activity in SOT as described in established exercise program recommendations. Website and video quality was determined using DISCERN, Global Quality Scale (GQS), and Patient Education Materials and Assessment Tool (PEMAT; threshold for which material is deemed understandable or actionable is >70%). Parametric and non-parametric tests were used to assess website and video characteristics, content, and quality metrics.

**Results:**

Forty-nine unique SOT websites (*n* = 15) and videos (*n* = 34) were identified, with the two most common categories being foundation/advocacy organizations and scientific resources. The average reading grade level of websites was 13 ± 3. Website and video content scores varied significantly (websites 11.3 ± 6.4; videos 8.4 ± 5.3). DISCERN total score and GQS score were low (median range for DISCERN 2.5–3.0; median for GQS 2.0 for both websites and videos, out of 5). PEMAT understandability and actionability scores were also low across websites and videos (mean range 57%–67% and 47%–65%, respectively). Foundation/advocacy websites had higher content and quality scores compared to scientific organizations and news/media articles.

**Conclusions:**

To our knowledge, this is the first comprehensive assessment of online content and quality of website and video resources on physical activity and exercise in adult SOT recipients. There were a limited number of online English patient-directed resources related to physical activity in SOT, most of which only partly captured items outlined in consensus exercise program recommendations and were of low quality and understandability and actionability. This work provides important insight to the English-speaking transplant community on the current state of online exercise health information and provides future direction for resource development.

## Introduction

Solid organ transplantation (SOT) is a lifesaving procedure for many patients with end-stage organ failure, and also improves quality of life and physical function (1, 2). Physical activity has been shown to improve physical function, quality of life and all-cause mortality in SOT recipients. In addition, exercise in SOT can potentially mitigate secondary transplant-related conditions including cardiovascular risk factors, fatigue, osteoporosis, and muscle atrophy, and promote return to work and societal roles (3, 4). However, the post-transplant effects of extended hospital length of stay, prolonged physical inactivity, and immunosuppression are known to have significant adverse effects on exercise tolerance, skeletal muscle dysfunction, and cardiometabolic risk factors (3–8).

Exercise post-transplant has commonly been undertaken with in-person facility-based programs. However, home-based exercise programs and online resources (i.e., websites and videos) have emerged as a promising strategy in the SOT population to provide greater accessibility and to mitigate infectious risk with the COVID-19 environment (9–11). The Internet is commonly used as a source of health information as it allows easy and immediate access to health information (12). Google and YouTube are two of the most frequently visited search engines on the Internet (13); however, the accuracy and reliability of online health information varies widely based on previously published reports (14–16).

Established exercise recommendations endorsed by the Canadian Society of Transplantation were published in 2019 (4). These consensus guidelines were targeted primarily for healthcare professionals and may not translate to patients. Further, there is a lack of online resources provided and/or endorsed by transplant programs. Thus, many SOT recipients may rely on information from online resources for further guidance. Given the emergence from the COVID-19 pandemic and greater reliance on virtual communication, online resources in the form of websites and videos are more likely be utilized to provide education and instruction on rehabilitation modalities in SOT. However, the content and quality of these online resources has not been evaluated. Thus, the objectives of this study were: (1) to characterize websites and videos on exercise and physical activity in adult SOT in the English language; and (2) to assess the content, quality, and understandability and actionability of these online resources. We hypothesized that websites and videos on exercise and physical activity would be variable in their content and quality scores, with foundation/transplant websites and videos having higher content and quality scores compared to other categories across all transplant groups.

## Methods

### Search strategy

A search for websites and videos on exercise and physical activity in SOT was conducted on Google and YouTube using the English search term “exercise and physical activity in solid organ transplantation.” The video filter on Google was used to search for videos. The search term was designed to capture the range of exercise and physical activity resources available for SOT recipients online. A single search was performed on July 17, 2022, and websites and videos were saved for review. An updated search was conducted on September 3, 2023 to identify any new or relevant websites. A United States (US) internet protocol (IP) address and virtual private network was used following the removal of web browsers' history and cookies. A US IP address was used as the US has the largest transplant population globally (17).

### Study selection

The first 200 identified websites on Google, and 200 videos on Google (video filter) and YouTube were screened for eligibility in July 2022. For the updated search in September 2023, the first 50 websites and videos for each search engine were screened for eligibility. Websites and videos in the English language were included if they provided education or instruction on exercise or physical activity in adult SOT defined as structured activity aimed at improving physical fitness or health or any activity requiring skeletal muscle movement and increased energy expenditure, respectively (18). Websites and videos were excluded for any of the following reasons: (1) duplicate websites or videos; (2) non-English websites or videos; (3) websites or videos that require a fee to access; (4) websites and videos unrelated to exercise and physical activity in SOT; (5) scientific articles. A list of eligible websites and videos and their rank in each search engine are provided in Supplementary Materials Tables S1 and S2.

### Data abstraction

#### Website characterization

To characterize websites, the following information was abstracted from each website: rank on Google, URL (unform resource locator), geographic location, website category, readability, and SOT group (i.e., lung, kidney, heart, liver, all SOT groups). Websites were categorized as: foundation/transplant organizations, scientific resources, news/media program or industry/for profit. Website readability was evaluated using the Flesch Reading Ease Score (FRES) and the Flesch-Kincaid Grade Level from the built-in readability statistics function of Microsoft Word 2018^TM^, as further outlined in the Supplementary Material (19, 20).

#### Video characterization

The following data was abstracted for each eligible video if applicable: rank on Google or YouTube, title of the video, URL (uniform resource locator), source of video upload, country of origin (if available), and SOT group (i.e., lung, kidney, heart, liver, all SOT groups) (14, 15). Videos were categorized as foundation/transplant organizations, news/media program, industry/for profit, private medical professional-generated content, user-generated content. The number of views or “viewing rate” and the “interaction index” which represents the average number of views for the video per day and the extent to which viewers are engaging with the video, respectively were calculated as previously described (21):

∙ViewingRate=(#ofviews/#ofdayssinceupload)×100%

∙InteractionIndex=[(#oflikes−#ofdislikes)/(total#ofviews)]×100%

### Website and video evaluation

#### Website and video content

Website and video content was evaluated based on a predefined scoring system of 25 key components of exercise and physical activity in SOT, as adapted from previously established exercise recommendations endorsed by the Canadian Society of Transplantation [Table 1; definition, short and long-term benefits, exercise training, FIT (frequency, intensity, time), safety considerations, educational aspects of exercise training in SOT] (4). The content table was also reviewed by members of our team who were instrumental in creating these exercise program recommendations, as well as patient partners. Content was scored as “yes” (1) indicating the item was addressed or “no” (0) indicating the item was not addressed for the 25 items listed in Table 1.

**Table 1:** Criteria for content scoring of exercise and physical activity in SOT websites and videos.

Category	Criteria	Other examples	Website (*n* = 15)	Video (*n* = 34)
Definition (/1)	Exercise or physical activity	Structured activity that is aimed at improving physical fitness or health, or any activity that requires skeletal muscle movement and increases energy expenditure	1 (7%)	3 (9%)
Short and long-term benefits (/6)	Physical function Quality of life Muscle strength Cardiometabolic Risk Factors Osteoporosis Fatigue	Physical health, exercise capacity, tolerance, fitness, endurance, stamina, ability to exercise Daily function, well-being, mental well-being, depression, anxiety Muscle conditioning, muscle atrophy Hypertension, hyperlipidemia, diabetes, overweight/obesity, cardiovascular disease Bone density, strengthen bone Tiredness, energy levels	14 (93%) 13 (87%) 12 (80%) 10 (67%) 6 (40%) 4 (27%)	18 (53%) 18 (53%) 14 (41%) 13 (38%) 7 (21%) 7 (21%)
Exercise training (/4)	Endurance/aerobic training Resistance/strength training Stretching/flexibility exercises Balance exercises	Cardiovascular activity, walking (ground-based or on a treadmill), cycling (stationary or outdoor), swimming, running, rowing, Nordic walking, etc. (+/– interval training) Training with free weights, resistance bands, resistance machine or body weight, etc. (+/– interval training) Thoracic mobility, upper and lower extremity	13 (87%) 10 (67%) 6 (40%) 2 (13%)	23 (68%) 27 (79%) 12 (35%) 6 (18%)
FIT^a^ (/3)	Frequency Intensity Time	Exercise progression	9 (60%) 10 (67%) 7 (47%)	20 (59%) 16 (47%) 25 (74%)
Safety considerations (/5)	Exercise training safety recommendations Monitoring exertional tolerance Medication use Fall risk Other	Consult with your doctor, caregiver/partner in home environment, exercise safety screening tool (i.e., PAR-Q+), Heart rate, perceived exertion scale (i.e., BORG) Effect of post-transplant medications (i.e., steroids, calcineurin inhibitors) Loss of balance Cardiovascular (hypotension, dizziness, cardiac issues), musculoskeletal (arthritis, low back pain, acute injury, overuse injury), diabetic complications (hypoglycemia), comorbidities	8 (53%) 6 (40%) 7 (47%) 1 (7%) 7 (47%)	19 (56%) 8 (24%) 8 (24%) 0 (0%) 6 (18%)
Educational aspects of exercise training in SOT (/6)	Goal setting Equipment Sources of Motivation Considerations if graft dysfunction Consideration of exercise and infection/post-hospitalization Other	Equipment to exercise (i.e., treadmill, stationary bike, bands, weights), equipment to monitor activity (smart watch, mobile app), footwear Family/friends, technology Organ rejection (acute or chronic) Hydration, sun protection, weather, air quality health index	4 (27%) 6 (40%) 5 (33%) 3 (20%) 2 (13%) 3 (20%)	4 (12%) 12 (35%) 8 (24%) 0 (0%) 2 (6%) 8 (24%)
Overall (/25)			11.3 ± 6.4	8.4 ± 5.3

FIT, frequency, intensity, time; SOT, solid organ transplantation.

Data are shown as proportions, *n* (%) or mean ± standard deviation.

Website content was scored a “yes” (1) indicating the item was addressed or “no” (0) indicating the item was not addressed.

^a^ FITT principle: type is captured under the content item “Exercise Training” as additional examples.

#### Reliability and quality

The reliability and quality of websites and videos were assessed using the modified DISCERN score (Supplementary Material Table S3), adapted from the original DISCERN tool used for health information (22), and the Global Quality Scale (GQS; Supplementary Material Table S4) (23). The modified DISCERN score is a 5-point scale that assesses clear website and video aims, reliability of sources, unbiased information, availability of references and areas of uncertainty (22). The Global Quality Scale (GQS) is a 5-point scale that assesses website and video accessibility, quality, overall flow of information, and the usefulness of websites or videos to a patient (23). Therefore, higher scores on the DISCERN and GQS scale indicate higher quality (22, 23).

#### Understandability and actionability

Understandability and actionability of online resources were evaluated using the validated Patient Education Materials and Assessment Tool (PEMAT) Printable and Audio/Visual Materials, respectively (24). PEMAT is designed as a guide to determine whether patients can understand and act on the information provided. Understandability refers to the ability of individuals from diverse backgrounds and varying health literacies to process, comprehend, and communicate key messages of patient educational materials (24). On the other hand, actionability reflects whether individuals can act on patient education materials (24). The understandability of websites and videos was assessed using a 17 and 13-point scale, respectively, that evaluates content, word choice and style, organization, layout and design, and use of visual aids. The applicability of websites and videos was determined using a 6 and 4-point scale, respectively (Supplementary Material Tables S5 and S6) (24). The threshold for which material is deemed understandable or actionable is >70%, as defined by the PEMAT tool (24).

### Agreement between content and quality measurements

The content and quality of websites and videos were evaluated by a primary reviewer (TD). The secondary reviews were conducted by three independent reviewers (RM, NAK, MM). For the overall website and video content score, DISCERN score, PEMAT score and GQS score, discrepancies were addressed with a consensus score between the primary and secondary reviewers. More significant discrepancies were assessed by a fourth reviewer with expertise in exercise and physical activity in SOT (DR).

### Statistical analysis

Descriptive statistics were used to characterize available website and video resources. Between-group differences across website and video characterization and evaluation criteria were assessed using chi-square, Fisher exact, Wilcoxon rank sum or Kruskal-Wallis tests as appropriate. The inter-rater reliability for the overall DISCERN and GQS score was calculated with Cohen's Kappa. Statistical analyses were performed using SPSS (version 23.0; IBM, Armonk, NY, USA), with two-tailed *p* < 0.05 considered to be significant.

## Results

### Website and video characteristics

**Figure 1:**
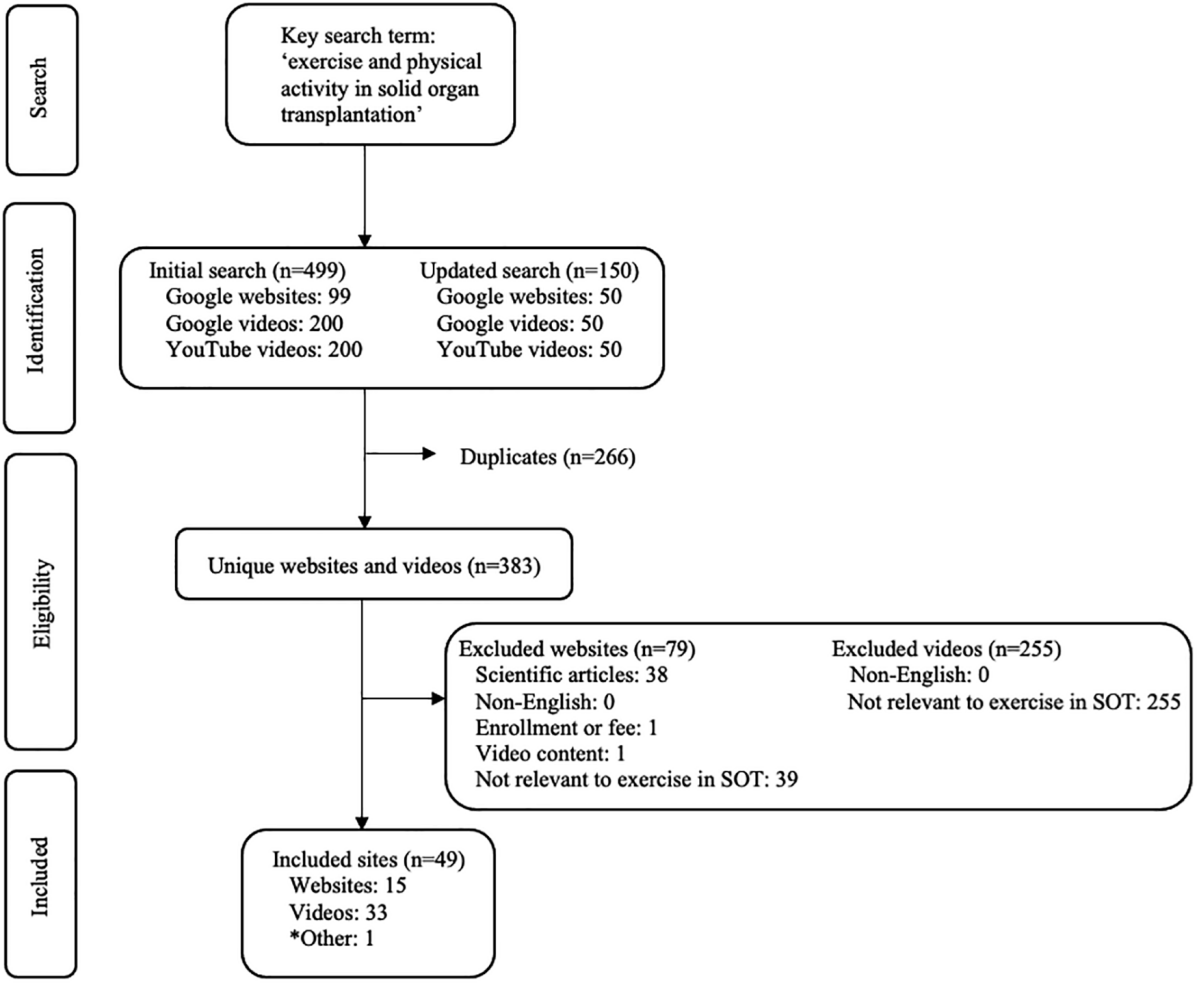
Flow diagram of the search results and study selection. *Other: exercise and physical activity in SOT video identified directly through additional resources from eligible resources. SOT, solid organ transplantation.

A total of 49 unique online English resources for SOT recipients were identified (Figure 1; *n* = 15 websites and *n* = 34 videos). A list of the eligible websites and videos and their rank in each search engine are provided in Supplementary Material Tables S1 and S2. Website and video characteristics are summarized in Table 2.

**Table 2:** Website and video characteristics.

Characteristics	Websites (*n* = 15)	Videos (*n* = 34)	*p*-value
Website/video category^a^ Foundation/transplant organization Scientific organization News/media Industry/for-profit Private medical-professional User-generated content	6 (40%) 6 (40%) 3 (20%) 0 (0%) – –	17 (50%) 8 (24%) 0 (0%) 0 (0%) 2 (6%) 7 (20%)	–
Continent of origin North America Australia Asia Europe/United Kingdom	7 (47%) 4 (27%) 2 (13%) 2 (13%)	19 (56%) 2 (6%) 4 (12%) 9 (26%)	0.20
Target population All SOT types Heart and/or lung Kidney Liver	12 (79%) 2 (14%) 1 (7%) 0 (0%)	15 (44%) 4 (12%) 12 (35%) 3 (9%)	0.07
Flesch-Kincaid grade level	13.0 ± 2.7	–	–
Viewing rate	–	41.9 [10.3–76.0]	–
Interaction index	–	1.6 [0.0–3.3]	–
Content total score (0–25)	11.3 ± 6.4	8.4 ± 5.3	0.11
Modified DISCERN total score (1–5)	3.0 [2.0–3.0]	2.5 [1.0–4.0]	0.86
Global quality scale (1–5)	2.0 [2.0–4.0]	2.0 [1.75–3.0]	0.18
PEMAT understandability score (1%–100%)	56.6% ± 21.3%	67.0% ± 16.3%	0.11
PEMAT actionability score (1%–100%)	46.7% ± 39.9%	65.0% ± 33.1%	0.10

PEMAT, patient education materials and assessment tool; SOT, solid organ transplantation.

Data are shown as proportions, *n* (%), mean ± standard deviation, and median [interquartile range].

^a^ Parametric (independent *t*-test) and non-parametric (chi-square) tests were used to assess website and video characteristics. Between-group differences not available for category as different categories across websites and videos.

The two most common categories were foundation/transplant organizations (websites: 40%, videos: 50%) and scientific resources (websites 40%, videos: 24%). Most websites and videos on physical activity in SOT captured all types of SOT groups (websites: 79%, videos: 44%), whereas others focused on specific transplant types including heart and/or lung (websites: 14%, videos: 12%), kidney (websites: 7%, videos: 35%), and liver (websites: 0%, videos: 9%). The average reading grade level of SOT websites was equivalent to grade 13 (advanced skill at college or university), corresponding to a difficult readability level. The median video viewing rate and interaction index were low (41.9% IQR [10–76] and 1.6% IQR [0–3], respectively). There were no significant differences in content, quality, and PEMAT understandability or actionability scores between websites and videos, as shown in Table 2.

#### Website content

**Figure 2:**
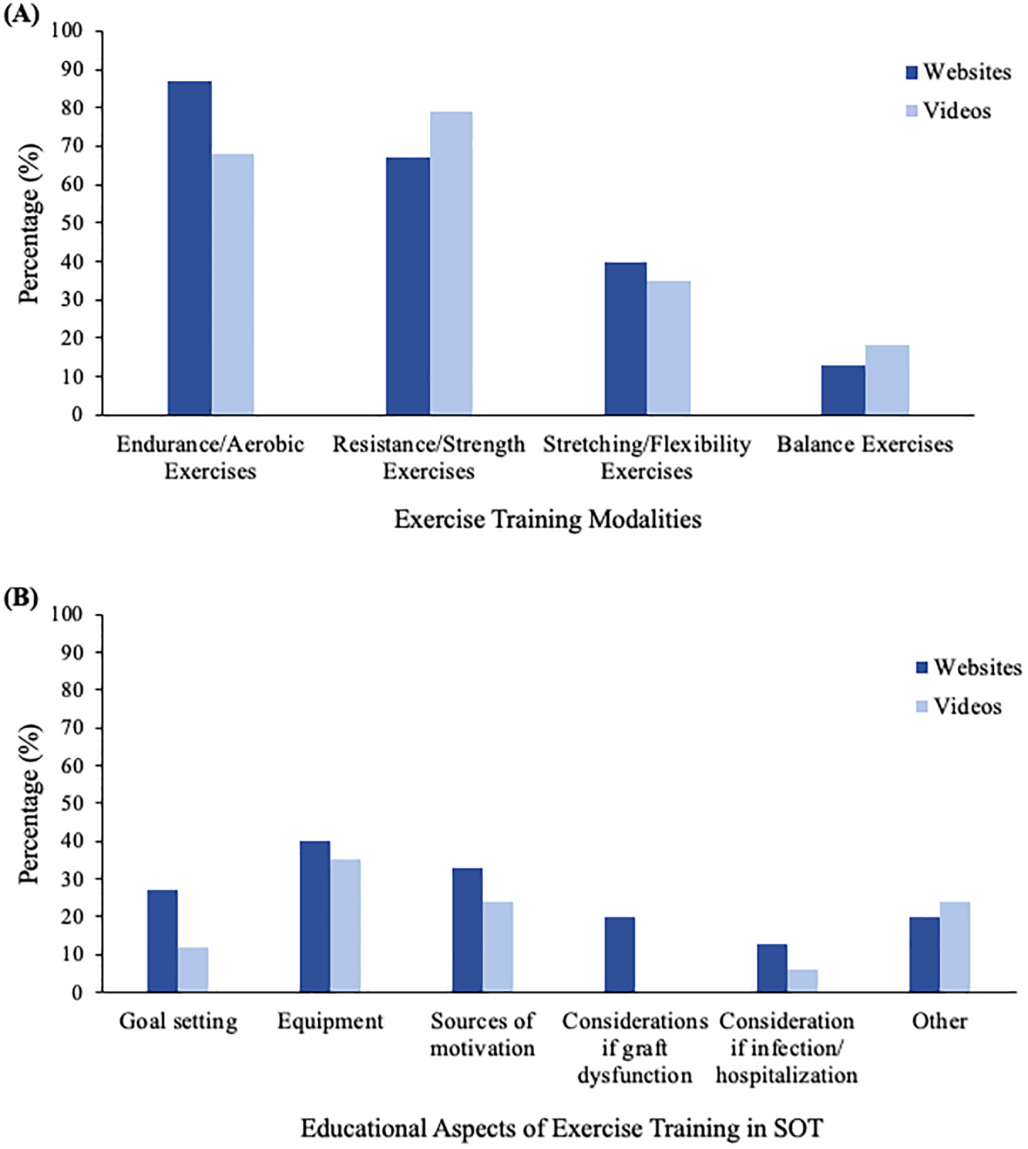
Percentage of websites and videos that discussed (**A**) exercise training modalities and (**B**) educational aspects of exercise training in SOT. *Other: hydration, sun protection, weather, air quality health index. SOT, solid organ transplantation.

There was significant heterogeneity in content across websites. The average total content score across websites was 11.3 ± 6.4 out of 25, as shown in Table 2. The majority of websites discussed the short and long-term benefits of physical activity, with less emphasis on osteoporosis management (40%) and fatigue (27%). Most websites focused on aerobic (87%) and resistance (67%) training, with fewer websites describing flexibility (40%) and balance (13%) exercises, as shown in Figure 2A. Some safety considerations were addressed such as medication use (47%), cardiovascular, musculoskeletal, and diabetic complications (47% for all three), whereas falls risk was only highlighted in one website (7%). Similarly, educational components as they relate to equipment needs (40%), motivation (33%), goal setting (27%), and considerations for exercise in the setting of graft dysfunction (20%) were less commonly addressed, Figure 2B. Website content was different across website categories (*p* = 0.001), with more comprehensive content scores across foundation/advocacy websites (content score 17.0 ± 4.6 out of 25) as compared to scientific organizations (content score 9.5 ± 2.7, *p* = 0.01) or news/media articles (3.3 ± 3.2, *p* = 0.001; Supplementary Material Table S7).

#### Video content

Content varied significantly across the 34 videos with an average total content score of 8.4 ± 5.3 out of 25, as shown in Table 2. Approximately half of the videos discussed the short and long-term benefits of physical activity, but did not emphasize muscle strength (41%), cardiometabolic risks (38%), osteoporosis (21%), or fatigue (21%). Similar to websites, most videos focused on traditional exercise training modalities including aerobic (68%) and resistance (79%) training, with less emphasis on flexibility (35%) and balance (18%) exercises (Figure 2A). Only a minority of videos discussed safety (monitoring of exertional tolerance 24%, medication use 24%, cardiovascular, musculoskeletal, or diabetic complications/comorbidities 18% for all three, and 0% addressed falls risk). Demonstration of educational components were also limited across most videos including equipment (35%), motivation (24%), goal setting (12%), and considerations for exercise in the setting of infection/post-hospitalization (6%) and graft dysfunction (0%), Figure 2B. Video content was not different across video categories (Supplementary Material Table S8).

#### Website and video quality

Website quality (DISCERN 3 IQR [2–3], GQS 2 [IQR 2–4] out of 5) and video quality (DISCERN 2.5 IQR [1–4]; GQS 2 IQR [1.75–3]) were both low. The scores for individual DISCERN and GQS questions are shown in Supplementary Material Tables S3 and S4, respectively. The presentation of balanced and unbiased information was frequent (websites: 93%; videos: 79%), while addressing additional sources of information listed for patient reference and areas of uncertainty surrounding physical activity in SOT was infrequent (websites: 40% for both; videos: 21% and 38%, respectively). The median DISCERN total score and GQS score varied across website categories, with higher scores for foundation/advocacy websites compared to news/media articles (DISCERN *p* = 0.01; GQS *p* = 0.004, Supplementary Material Table S7). Median DISCERN and GQS total scores were not different across video categories (Supplementary Material Table S8).

The inter-rater reliability for the overall median DISCERN and GQS scores for both websites and videos was very good to excellent, as shown in the Supplementary Material Results.

#### Website understandability and actionability

PEMAT understandability and actionability scores were low across websites (57% ± 21% and 47% ± 40%, respectively; threshold for understandable or actionable materials is >70%). Scores for individual PEMAT understandability and actionability questions are shown in Supplementary Material Table S5. Understandability scores were highest in terms of website content being broken down into short sections including presence of informative headings and presenting information in a logical sequence. Actionability score was highest for websites that identified at least one action the users can take, whereas lack of visuals such as tables or diagrams resulted in lower scores. PEMAT understandability score varied across website categories (*p* = 0.02), with higher scores for foundation/advocacy websites (PEMAT understandability score 70.7% ± 16.0%) compared to news/media articles (PEMAT understandability score 31.9% ± 6.4%; *p* = 0.02). PEMAT actionability scores were not significantly different across website categories (Supplementary Material Table S7).

#### Video understandability and actionability

PEMAT understandability and actionability scores were low across videos (67% ± 16% and 65% ± 33%, respectively; threshold for understandable or actionable materials is >70%). Scores for individual PEMAT understandability and actionability questions are shown in Supplementary Material Table S6. Similar to websites, PEMAT understandability scores were highest for organizational aspects which presented the information in brief sections with a logical sequence. The lowest scores for understandability were observed when summaries, visual aids and/or simple tables were not utilized. PEMAT actionability scores were highest for videos that identified at least one action that users could take, whereas the lowest scores for action were observed when explicit action steps, tables or diagrams were not available. PEMAT understandability and actionability scores did not significantly differ across video categories (Supplementary Material Table S8).

## Discussion

To our knowledge, this is the first study to characterize online resources in English on exercise and physical activity in SOT, and to assess the content, quality, understandability and actionability of patient-directed online resources for SOT recipients. Increasing awareness of and accessibility to high-quality online resources on physical activity and exercise in SOT may help promote engagement and adherence to activity post-transplant.

Content scores related to exercise and physical activity in SOT were low across websites and videos, and only minimally captured items outlined in consensus exercise programs (4). These results are similar to a study evaluating the content of online information on physical activity in osteoporosis, which found that the majority of webpages presented information inconsistent with established exercise guidelines (25). According to a qualitative study of the perspectives of healthcare professionals on digital health interventions to support physical activity in SOT, a suitable intervention should include features that promote self-management including physical activity monitoring, tailored education, and access to behavioural change strategies (26). However, only a minority of websites and videos discussed safety considerations and educational aspects of exercise and physical activity. Safety recommendations should consider monitoring of exertional tolerance, exercise in the setting of graft dysfunction or illness, as well as appropriate hydration, sun protection, and consideration of air quality. Further, online interventions that consider behavior change elements, such as goal setting and motivational practices, have also been shown to be particularly effective in promoting self-management and adherence to physical activity. As such, our findings demonstrate that most online resources did not include the key components highlighted across SOT exercise training consensus guidelines, which is consistent with other studies in chronic diseases (14–16, 25, 27, 28).

It is often difficult to assess the quality of online resources on the internet. Information presented on the internet often lacks regulatory oversight and is not subjected to the rigors of quality assurance which increases the risk of disseminating inaccurate information (29, 30). Website and video quality on exercise and physical activity were low. The majority of websites and videos did not use reliable sources of information, lacked additional resources for patient reference, and did not mention areas of uncertainty, which is consistent with previous studies (14–16). This could potentially be remedied by using reliable sources and providing additional online resources for patients seeking further information.

Online materials for exercise and physical activity in SOT also demonstrated low understandability and actionability domains. A minimum threshold of 70% is required for patient-education materials to be considered understandable and/or actionable; however, only 41% of websites and videos met the threshold for understandability and actionability. Of these, only 29% met the threshold for both understandability and actionability. These scores are similar to those reported in other chronic diseases, highlighting an opportunity to improve understandability and actionability across patient educational materials (31–33). Understandability could be improved with the inclusion of short summarized key messages as well as visual aids, which can also serve a dual purpose in improving health literacy. Actionability can be improved by breaking down actions into explicit, manageable steps and providing tangible tools, including exercise templates and charts. Future online resources should consider improving understandability and actionability scores to promote self-management and adherence to exercise and physical activity post-transplant.

Transplant patients are unique in that they are often followed closely by their transplant programs. It is important for healthcare providers to be aware of and to direct their patients to high-quality patient-directed resources. Foundation/advocacy websites and videos had higher content, DISCERN and GQS scores compared to other category types. The online resources that received the highest scores in terms of content, quality, and understandability and actionability were the Canadian Network for Rehabilitation and Exercise for Solid Organ Transplant Optimal Recovery (CAN-RESTORE) and the World Transplant Games Federation- Refit for Life (34, 35). Both online resources are national organizations with multidisciplinary input that provide comprehensive content on physical activity in SOT, tangible exercise action plans, tailored education, and inclusion of behavioural change strategies.

Health literacy plays an important role in self-efficacy and in the management of chronic diseases. To improve comprehension of health information, the American Medical Association (AMA) and National Institutes of Health (NIH) recommend online health information to be written at a grade 6 reading level. The average English reading level of SOT websites was equivalent to grade 13 (advanced skill at college or university), with no websites written at a grade 6 level or lower, highlighting an opportunity to improve comprehension among the SOT community. These results are similar to a meta-analysis analyzing the readability of physical activity educational resources, which found that resources with physical activity-related content had poor readability indices which exceeded the 10th grade reading level (36). Several transplant studies have noted low health literacy among SOT recipients (37). Low health literacy is associated with reduced treatment adherence, increased burden of chronic disease, and poor health outcomes (38). This is particularly applicable to the field of transplantation as the long-term success of transplant patients is predicated on self-management, including adherence to exercise and physical activity, which has been shown to reduce the risk of all-cause mortality and improve health-related quality of life (39, 40). Houts et al. reviewed the role of pictures in improving communication on health education and found that the inclusion of pictures and/or diagrams improved attention and comprehension of health information, and may improve adherence (41). Similarly, the inclusion of summaries with key takeaway points has also been shown to improve comprehension and adherence to health information (42, 43). Thus, further work to improve health literacy in patient-education materials may promote self-efficacy and management in SOT, which may translate to improved outcomes post-transplant.

The results of this study should be interpreted considering the following limitations. First, the searches were restricted to websites and videos published in English and limited to the first 200 hits each on Google and YouTube. However, studies have shown that most individuals typically examine the first page of a search engine when searching for online information, suggesting that most (if not) all patient-accessed resources were captured within our search (44). Second, we used a US Internet Protocol address for our search; therefore, search results may be different if the search was conducted in other countries. Third, there are no gold-standard reference guidelines for exercise-training in solid organ transplant recipients, therefore it is possible that we may not have captured all elements across our predefined content scoring system. However, the content score was developed using consensus guidelines, as well as the expertise across our multidisciplinary SOT group and patient partners, which will be a valuable framework for future studies. Fourth, although there was only one primary reviewer, there were three different secondary reviewers which poses the risk of evaluation bias; however, this limitation was minimized through standardization of assessment training, discrepancy review, and requirement for consensus to be reached between the primary and secondary reviewers. Despite these limitations, this is the first comprehensive assessment of patient-directed online health information resources on physical activity and exercise in SOT recipients that included the input of patient partners.

## Conclusion

In conclusion, there are a limited number of English resources on exercise and physical activity in SOT. Exercise and physical activity content only partly captured items outlined in consensus exercise program recommendations with a notable limitation of safety and educational components mentioned. Websites and videos were observed to be of low quality, understandability and actionability. This study demonstrates the need for targeted strategies that consider health literacy, as well as comprehensive content and actionability in developing exercise and physical activity resources for English speaking SOT recipients.

## Data Availability

The raw data supporting the conclusions of this article will be made available by the authors, without undue reservation.
