# Supplementary material for: Online resources as a source of information for exercise and physical activity in solid organ transplant recipients
Source: Front Sports Act Living. 2024 Apr 30;6:1353663. doi: 10.3389/fspor.2024.1353663 (PMC11091340; doi:10.3389/fspor.2024.1353663)
Supplement: Supplementary file 1 [file Datasheet1.docx]

Supplementary Material

**ONLINE RESOURCES AS A SOURCE OF INFORMATION FOR EXERCISE AND PHYSICAL ACTIVITY IN SOLID ORGAN TRANSPLANT RECIPIENTS**

Tania Da Silva, MSc^1,2^; Rozhan Momen, MSc^2,3^; Noor Al Kaabi, MSc^2,4^; Muhib Masrur, MSc^2^; Sandra Holdsworth, Dip^5^; Karina Prevost, BSc^5^; Sherrie Logan, BSc, BA^5^; Daniel Santa Mina, PhD^2,3^; Istvan Mucsi, MD, PhD^6^; Mamatha Bhat, MD, PhD^6^; Ana Carolina Alba, MD, PhD^6,7^; W. Darlene Reid, PhD^8^; Manoela Ferreira, PhD^5,8^; Sunita Mathur, PhD^5,8^; Tania Janaudis-Ferreira, PhD^5,9,10^; Lisa Wickerson, MSc, PhD^2,5,6,8,^; Dmitry Rozenberg, MD, PhD^2,5,6,11^

^1^Temerty Faculty of Medicine, University of Toronto, Toronto, Ontario, Canada
^2^Toronto General Hospital Research Institute, University Health Network, Toronto, Ontario, Canada
^3^Faculty of Kinesiology and Physical Education, University of Toronto, Toronto, Ontario, Canada
^4^Institute of Medical Science, University of Toronto, Ontario, Canada
^5^Canadian Donation and Transplantation Research Program, Edmonton, Alberta, Canada
^6^Ajmera Transplant Program, University Health Network, Toronto, Ontario, Canada
^7^Ted Rogers Centre for Heart Research, Toronto, Ontario, Canada
^8^Department of Physical Therapy, Temerty Faculty of Medicine, University of Toronto, Toronto, Ontario, Canada
^9^School of Physical and Occupational Therapy, McGill University, Montreal, Quebec, Canada
^10^Respiratory Epidemiology and Clinical Research Unit, Centre for Outcomes Research and Evaluation, Research Institute of the McGill University Health Centre, Montreal, Quebec, Canada
^11^Division of Respirology, Temerty Faculty of Medicine, University Health Network, University of Toronto, Ontario, Canada

**Supplemental Material**

**Supplementary Methods**

**Supplementary Results**

**Supplementary Table 1.** Website uniform resource identifier.

**Supplementary Table 2.** Video uniform resource identifier.

**Supplementary Table 3.** Modified DISCERN score.

**Supplementary Table 4.** Global Quality Scale.

**Supplementary Table 5.** Patient Education Material and Assessment Tool for Printable Materials.

**Supplementary Table 6.** Patient Education Material and Assessment Tool for Audio/Visual Materials.

**Supplementary Table 7.** Website content, quality, and understandability and actionability across website categories.

**Supplementary Table 8.** Video content, quality, and understandability and actionability across video categories.

**Supplementary Methods**

*Flesch Reading Ease Score [FRES] and the Flesch-Kincaid grade level*

The FRES determines comprehension difficulty of written information, with lower scores indicating greater difficulty (0–30, very difficult; 30–50, difficult; 50–60, fairly difficult; 60–70, standard; 70–80, fairly easy; 80–90, easy; 90–100, very easy) (1). The Flesch-Kincaid Grade Level calculates a readability score equivalent to a United States grade level, with lower scores indicating the material is easier to read (2).

**Supplementary Results**

*Website Quality*

The inter-rater reliability (Cohen’s kappa) for the overall median DISCERN score was 0.82 (95% CI [0.70, 0.93]; 87% agreement across websites), with 100% consensus reached between the two reviewers. The inter-rater reliability (Cohen’s kappa) for the median Global Quality Score (GQS) score was 0.91 (95% CI [0.83, 1.00]; 93% agreement across videos with initial independent assessment), with 100% consensus reached between the two reviewers.

*Video Quality*

The inter-rater reliability (Cohen’s kappa) for the overall median DISCERN score was 0.78 (95% CI [0.70, 0.86]; 82% agreement across videos). The inter-rater reliability (Cohen’s kappa) for the median GQS score was 0.88 (95% CI [0.81, 0.94]; 91% agreement across videos with initial independent assessment), with 100% consensus reached between the two reviewers.

**Supplementary Table 1:** Website uniform resource identifier.

| Website uniform resource identified | Rank Google® |
| --- | --- |
| https://exerciseright.com.au/organ-transplantation | 1 |
| http://exerciseismedicine.com.au/wp-content/uploads/2018/06/EIM-fact-sheet-template_organ-transplantation.pdf | 20 |
| https://wtgf.org/library/ | 21 |
| https://www.edqm.eu/documents/52006/75920/exercice-your-way-to-better-post-transplant-health-july-2016.pdf/c5c928b6-a191-1916-ad55-a85319f8d0a4?t=1623404904278 | 22 |
| https://transplant.org.au/living-with-your-transplant/post-transplant-fitness/being-active-with-your-transplant/ | 28 |
| https://now.aapmr.org/solid-organ-transplant-rehabilitation/ | 32 |
| https://www.astellas.com/th/en/news/8621 | 33 |
| https://www.acc.org/latest-in-cardiology/articles/2021/02/12/13/33/regaining-cardiorespiratory-fitness-post-heart-transplantation | 34 |
| http://isn-iran.ir/images/uploaded/national%20congress%2095/%D9%BE%DB%8C%D9%  88%D9%86%D8%AF%2097/x%20dr%20poorgharib-REHABILITATION%20FOR%20S OLID%20ORGAN.pdf | 37 |
| https://hartfordhospital.org/File%20Library/Services/Transplant%20Services/Symposium/2017-Lifestyle-Medicine.pdf | 38 |
| https://www.mayoclinic.org/tests-procedures/kidney-transplant/about/pac-20384777 | 39 |
| https://www.itnnews.co.in/indian-transplant-newsletter/issue57/Fitness-for-transplant-recipients-936.htm | 45 |
| https://canrestore.wordpress.com/for-transplant-patients-caregivers/ | 59 |
| https://stanfordhealthcare.org/medical-treatments/h/heart-transplant/what-to-expect/exercise.html | 62 |
| https://theconversation.com/living-and-competing-after-an-organ-transplant-92195 | 66 |

**Supplementary Table 2:** Video uniform resource identifier.

| Video uniform resource identified | Rank YouTube® | Rank Google Video® |
| --- | --- | --- |
| https://www.youtube.com/watch?v=dFvFJwXgRjs&ab_channel=NationalCentrefor  SportandExerciseMedicine | 1 |  |
| https://www.youtube.com/watch?v=WIJDhSr7uEU | 2 |  |
| TheCentreforLivingOrganDonationatUHN | 3 |  |
| https://www.youtube.com/watch?v=sWoDaOWYnw4&ab_channel=  CanadianDonationandTransplantResearchProgram | 5 |  |
| https://www.youtube.com/watch?v=bwIMzWULwLo&t=767s&ab_channel=World  TransplantGamesFederation | 6 |  |
| https://www.youtube.com/watch?v=b_p5bmPeq9k&ab_channel=UWMedicine | 7 |  |
| https://www.youtube.com/watch?v=pC5BwwONH0c&t=359s&ab_channel=World  TransplantGamesFederation | 8 |  |
| https://www.youtube.com/watch?v=9jnTc_PaSgk&ab_channel=JimJablonski | 9 |  |
| https://www.youtube.com/watch?v=YGQW8aJO3QQ&ab_channel=TravaneMorrison | 10 |  |
| https://www.youtube.com/watch?v=KpWc8zOSyUY&ab_channel=AcademyofKidney  DiseasesandTransplantation | 13 |  |
| https://www.youtube.com/watch?v=5QTGlyCjXVw&ab_channel=CindyFlores | 14 |  |
| https://www.youtube.com/watch?v=TrOIC3_T-U8&ab_channel=MichiganMedicine | 17 |  |
| https://www.youtube.com/watch?v=stQBD2kPdq8&ab_channel=UHNPatientEducation | 19 |  |
| https://www.youtube.com/watch?v=acFsqewZenI&ab_channel=StanfordHealthCare | 20 |  |
| https://www.youtube.com/watch?v=3CdiN2XsHbs&ab_channel=NationalKidney  FoundationofIllinois | 25 |  |
| https://www.youtube.com/watch?v=MbeP-1DFrYo&ab_channel=DrSanjeev  GulatiKidneyClinic | 26 |  |
| https://www.youtube.com/watch?v=yI-JNkkZKFI&ab_channel=Vammaisurheilu | 37 |  |
| https://www.youtube.com/watch?v=OlibjAWDUyw&ab_channel=PTReviewer | 52 |  |
| https://www.youtube.com/watch?v=En3-vdi-yuQ&ab_channel=Vammaisurheilu | 68 |  |
| https://www.youtube.com/watch?v=rQjXV0ac3v0&ab_channel=AcademyofKidney  DiseasesandTransplantation | 78 |  |
| https://www.youtube.com/watch?v=Vuz8x5NkmcE&ab_channel=TRIOTransplant  PresentationLibrary | 85 |  |
| https://www.youtube.com/watch?v=VNbKo6DYmvA&ab_channel=GeorgiaTransplant  Foundation | 118 |  |
| https://www.youtube.com/watch?v=hpDukmGvL1s&ab_channel=TransplantAustralia  OfficialAccount | 126 |  |
| https://www.youtube.com/watch?v=MMWyUo5RNwg&ab_channel=TravaneMorrison | 136 |  |
| https://www.youtube.com/watch?v=_qo0MliST_I&ab_channel=FitnessAndRehabIndia | 159 |  |
| https://www.youtube.com/watch?v=yaqk_vd9FqE&ab_channel=Olivia | 176 |  |
| https://www.youtube.com/watch?v=UibDZ16XglI&ab_channel=TheCentrefor  LivingOrganDonationatUHN |  | 7 |
| https://www.youtube.com/watch?v=kWK2seudthI&ab_channel=TheCentrefor  LivingOrganDonationatUHN |  | 21 |
| https://wtgf.org/refitforlife/guided-a-strength-mobility-and-flexibility-series/# |  | 25 |
| https://www.youtube.com/watch?v=UjwAwsbysHE | 12 |  |
| https://www.youtube.com/watch?v=thDzqPkmkFE&ab_channel=BSLMCEducators | 33 |  |
| https://www.youtube.com/shorts/zHSP_talgWg | 43 |  |
| https://www.youtube.com/watch?v=bzYGxwQNKT8&pp=ygU9ZXhlcmNpc2UgYW5kIHB  oeXNpY2FsIGFjdGl2aXR5IGluIHNvbGlkIG9yZ2FuIHRyYW5zcGxhbnRhdGlvbg%3D%3  D&ab_channel=Vammaisurheilu | 48 |  |
| https://transplant.org.au/fit-for-life/ |  |  |

**Supplementary Table 3:** Modified DISCERN score.

| Modified DISCERN score | Score | Websites  (n=15) | Videos  (n=34) |
| --- | --- | --- | --- |
| Reliability of information |  |  |  |
| 1. Are the aims clear and achieved? | Yes= +1 point, No= 0 point | 7 (47%) | 26 (77%) |
| 2. Are reliable sources of information used? | Yes= +1 point, No= 0 point | 7 (47%) | 15 (44%) |
| 3. Is the information presented balanced and unbiased? | Yes= +1 point, No= 0 point | 14 (93%) | 27 (79%) |
| 4. Are additional sources of information listed for patient reference? | Yes= +1 point, No= 0 point | 6 (40%) | 7 (21%) |
| 5. Are areas of uncertainty mentioned? | Yes= +1 point, No= 0 point | 6 (40%) | 13 (38%) |

Data are shown as proportions, n (%).

**Supplementary Table 4:** Global Quality Scale score.

| Score | Description | Websites  (n=15) | Videos  (n=34) |
| --- | --- | --- | --- |
| 1 | Poor quality, poor flow, most information missing, not useful for patients | 3 (20%) | 8 (24%) |
| 2 | Generally poor quality, poor flow, some information given but many important topics missing, of very limited use to patients | 6 (40%) | 12 (35%) |
| 3 | Moderate quality, suboptimal flow, some important information is adequately discussed but others poorly discussed, somewhat useful for patients | 2 (13%) | 11 (32%) |
| 4 | Good quality, good flow, most relevant information is covered but some topics not covered, useful for patients | 2 (13%) | 3 (9%) |
| 5 | Excellent quality, excellent flow, very useful for patients | 2 (13%) | 0 (0%) |

Data are shown as proportions, n (%).

**Supplementary Table 5:** Patient Education Material and Assessment Tool for Printable Materials for websites.

**Understandability**

| Item # Item Websites  (n=15) | | |
| --- | --- | --- |
| Topic: Content | | |
| 1 | The material makes the purpose completely relevant. | 7 (47%) |
| 2 | The material does not include information or content that distracts from its purpose. | 10 (67%) |
| Topic: Word Choice & Style | | |
| 3 | The material uses common, everyday language. | 0 (0%) |
| 4 | Medical terms are used only to familiarize audience with the terms. When used, medical terms are defined. | 11 (73%) |
| 5 | The material uses the active voice. | 10 (67%) |
| Topic: Use of Numbers | | |
| 6 | Numbers appearing in the material are clear and easy to understand. | 10 (67%) |
| 7 | The material does not expect the user to perform calculations. | 14 (93%) |
| Topic: Organization | | |
| 8 | The material breaks or “chunks” information into short sections. | 11 (73%) |
| 9 | The material’s sections have informative headers. | 11 (73%) |
| 10 | The material presents information in a logical sequence. | 11 (73%) |
| 11 | The material provides a summary. | 3 (20%) |
| Topic: Layout & Design | | |
| 12 | The material uses visual cues (e.g. arrows, boxes, bullets, bold, larger font, highlighting) to draw attention to key point. | 9 (60%) |
| Topic: Use of Visual Aids | | |
| 15 | The material uses visual aids whenever they could make content more easily understood (e.g. illustration of healthy portion size). | 3 (20%) |
| 16 | The material’s visual aids reinforce rather than distract from the content. | 3 (20%), N/A 7 (47%) |
| 17 | The material’s visual aids have clear titles or captions. | 5 (33%), N/A 7 (47%) |
| 18 | The material uses illustrations and photographs that are clear and uncluttered. | 7 (47%), N/A 7 (47%) |
| 19 | The material uses simple tables with short and clear row and column headings. | 2 (13%), N/A 13 (87%) |

Total Points**:** _____________
Total Possible Points**:** _____________
**Understandability Score (%):** _____________

(Total Points / Total Possible Points x 100)

*N/A= Not Applicable and will not be counted in the denominator.

**Actionability**

| Item # Item Websites  (n=15) | | |
| --- | --- | --- |
| 20 | The material clearly identifies at least one action the user can take. | 11 (73%) |
| 21 | The material addresses the user directly when describing actions. | 6 (40%) |
| 22 | The material breaks down any action into manageable, explicit steps. | 7 (47%) |
| 23 | The material provides a tangible tool (e.g. menu planners, checklists) whenever it could help the user take action. | 4 (27%) |
| 24 | The material provides simple instructions or examples of how to perform calculations. | 1 (7%), N/A 14 (93%) |
| 25 | The material explains how to use the charts, graphs, tables or diagrams to take actions. | 3 (20%), N/A 11 (73%) |

Total Points**:** _____________
Total Possible Points**:** _____________
**Actionability Score (%):** _____________
(Total Points / Total Possible Points x 100)

*N/A= Not Applicable and will not be counted in the denominator.
Data are shown as proportions, n (%).

**Supplementary Table 6:** Patient Education Material and Assessment Tool for Audio/Visual Materials for videos.

**Understandability**

| Item # Item Videos  (n=34) | | |
| --- | --- | --- |
| Topic: Content | | |
| 1 | The material makes the purpose completely relevant. | 25 (74%) |
| Topic: Word Choice & Style | | |
| 3 | The material uses common, everyday language. | 22 (65%) |
| 4 | Medical terms are used only to familiarize audience with the terms. When used, medical terms are defined. | 18 (53%) |
| 5 | The material uses the active voice. | 25 (74%) |
| Topic: Organization | | |
| 8 | The material breaks or “chunks” information into short sections. | 25 (74%) |
| 9 | The material’s sections have informative headers. | 19 (56%) |
| 10 | The material presents information in a logical sequence. | 32 (94%) |
| 11 | The material provides a summary. | 6 (18%) |
| Topic: Layout & Design | | |
| 12 | The material uses visual cues (e.g. arrows, boxes, bullets, bold, larger font, highlighting) to draw attention to key point. | 16 (47%), N/A (47%) |
| 13 | Text on the screen is easy to read. | 16 (47%), N/A (38%) |
| 14 | The material allows the user to hear the words clearly (e.g. not too fast, not garbled). | 28 (82%), N/A (3%) |
| Topic: Use of Visual Aids | | |
| 18 | The material uses illustrations and photographs that are clear and uncluttered | 11 (32%), N/A (56%) |
| 19 | The material uses simple tables with short and clear row and column headings | 3 (9%), N/A (68%) |

Total Points**:** _____________
Total Possible Points**:** _____________
**Understandability Score (%):** _____________
(Total Points / Total Possible Points x 100)

*N/A= Not Applicable and will not be counted in the denominator.

**Actionability**

| Item # Item Videos | | |
| --- | --- | --- |
| 20 | The material clearly identifies at least one action the user can take. | 31 (91%) |
| 21 | The material addresses the user directly when describing actions. | 20 (59%) |
| 22 | The material breaks down any action into manageable, explicit steps. | 16 (47%) |
| 25 | The material explains how to use the charts, graphs, tables or diagrams to take actions. | 2 (6%), N/A 85% |

Total Points**:** _____________
Total Possible Points**:** _____________
**Actionability Score (%):** _____________
(Total Points / Total Possible Points x 100)

*N/A= Not Applicable and will not be counted in the denominator.
Data are shown as proportions, n (%).

**Supplementary Table 7:** Website content, quality, and understandability and actionability across website categories.

| Questionnaire Items | Website category | | | | |
| --- | --- | --- | --- | --- | --- |
|  | **Overall (n=15)** | **Foundation/**  **Transplant Organization (n=6)** | **Scientific organization (n=6)** | **News/Media Article (n=3)** | **p-value** |
| Content total score | 11.3 ± 6.4 | 17.0 ± 4.6 | 9.5 ± 2.7 | 3.3 ± 3.2 | 0.001^†*^ |
| DISCERN score | 3.0 [2.0-3.0] | 3.5 [3.0-4.25] | 2.5 [1.75-3.0] | 1.0 [1.0-1.0] | 0.01^*^ |
| GQS score | 2.0 [2.0-4.0] | 4.0 [2.75-5.0] | 2.0 [2.0-2.25] | 1.0 [1.0-1.0] | 0.004^*^ |
| PEMAT understandability | 56.6% ± 21.3% | 70.7% ± 16.0% | 54.8% ± 19.8% | 31.9% ± 6.4% | 0.02^*^ |
| PEMAT actionability | 45.6% ± 38.6% | 70.8% ± 33.2% | 41.7% ± 40.8% | 8.3% ± 14.4% | 0.07 |

Data are shown as median [IQR] or mean ± SD.

P-value column represents one-way ANOVA or Kruskal–Wallis tests comparing each item across all website categories for normally distributed and non-normally distributed data, respectively. Industry/for-profit category not applicable for websites (n=0).

^†^P < 0.02 on pairwise comparison of foundation/transplant organization versus scientific organizations.
^*^P < 0.02 on pairwise comparison of foundation/transplant organization versus news/media article.

**Abbreviations:** GQS, Global Quality Scale; PEMAT, Patient Education Materials Assessment Tool.

**Supplementary Table 8:** Video content, quality, and understandability and actionability across video categories.

| Questionnaire Items | Video category | | | | | |
| --- | --- | --- | --- | --- | --- | --- |
|  | **Overall (n=34)** | **Foundation/ Transplant Organization**  **(n=17)** | **Scientific Organization**  **(n=8)** | **Private Medical-Professional (n=2)** | **User-Generated Content (n=7)** | **p-value** |
| Content total score | 8.4 ± 5.3 | 9.9 ± 5.8 | 7.8 ± 3.7 | 3.5 ± 3.5 | 6.9 ± 5.6 | 0.30 |
| DISCERN score | 3.0 [2.0-4.5] | 2.0 [1.0-3.75] | 4.0 [3.0-4.25] | 2.0 [2.0-2.0] | 1.0 [1.0-3.0] | 0.09 |
| GQS score | 2.0 [1.75-3.0] | 3.0 [2.0-3.0] | 2.0 [1.0-3.0] | 1.0 [1.0-1.0] | 2.0 [2.0-2.0] | 0.09 |
| PEMAT understandability | 65.0% ± 16.3% | 68.2% ± 14.6% | 68.9% ± 23.2% | 63.9% ± 19.6% | 62.7% ± 13.1% | 0.87 |
| PEMAT actionability | 65.0% ± 33.1% | 62.7% ± 33.7% | 63.5% ± 33.3% | 33.3% ± 47.1% | 81.0% ± 26.2% | 0.33 |

Data are shown as median [IQR] or mean ± SD.

P-value column represents one-way ANOVA or Kruskal–Wallis tests comparing each item across all video categories for normally distributed and non-normally distributed data, respectively. News/media program category not applicable for videos (n=0).

**Abbreviations:** GQS, Global Quality Scale; PEMAT, Patient Education Materials Assessment Tool.
